# Characterization and stabilization in process development and product formulation for super large proteinaceous particles

**DOI:** 10.1002/elsc.202000033

**Published:** 2020-07-19

**Authors:** Yanli Yang, Zhiguo Su, Guanghui Ma, Songping Zhang

**Affiliations:** ^1^ State Key Laboratory of Biochemical Engineering Institute of Process Engineering Chinese Academy of Sciences Beijing P. R. China

**Keywords:** characterization, denaturation, rational design, super large proteinaceous particles

## Abstract

Super large proteinaceous particles (SLPPs) such as virus, virus like particles, and extracellular vesicles have successful and promising applications in vaccination, gene therapy, and cancer treatment. The unstable nature, the complex particulate structure and composition are challenges for their manufacturing and applications. Rational design of the processing should be built on the basis of fully understanding the characteristics of these bio‐particles. This review highlights useful analytical techniques for characterization and stabilization of SLPPs in the process development and product formulations, including high performance size exclusion chromatography, multi‐angle laser light scattering, asymmetrical flow field‐flow fractionation, nanoparticle tracking analysis, CZE, differential scanning calorimetry, differential scanning fluorescence, isothermal titration calorimetry , and dual polarization interferometry. These advanced analytical techniques will be helpful in obtaining deep insight into the mechanism related to denaturation of SLPPs, and more importantly, in seeking solutions to preserve their biological functions against deactivation or denaturation. Combination of different physicochemical techniques, and correlation with in vitro or in vivo biological activity analyses, are considered to be the future trend of development in order to guarantee a high quality, safety, and efficacy of SLPPs.

**Abbreviations**
AAV: adeno‐associated virus
AF4: asymmetrical flow field‐flow fractionation
DLS: dynamic laser light scattering
DPI: dual polarization interferometry
DSC: differential scanning calorimetry
DSF: differential scanning fluorescence
EVs: extracellular vesicles
FMDV: foot and mouth disease virus
HBc: hepatitis B core antigen
HBs: hepatitis B surface antigen
HPSEC: high performance size exclusion chromatography
HPV: human papilloma virus
HRV: human rhinovirus
IEC: ion exchange chromatography
ITC: isothermal titration calorimetry
MALLS: multi‐angle laser light scattering
NTA: nanoparticle tracking analysis
SLPPs: super large proteinaceous particles
VLPs: virus like particles

## 1 INTRODUCTION

Virus, virus like particles (VLPs), and extracellular vesicles (EVs) are examples of super large proteinaceous particles (SLPPs) that have successful and promising applications in vaccination [1], gene therapy [2], and cancer treatment [3]. Although these bio‐particles are different, they present similar structural characteristics. They all have a large size that could range from 20 nm (adeno‐associated virus, AAV) to 1000 nm (measles virus) [4]. Furthermore, the particles often have a complex composition including a particle structure assembled by multiple structural proteins, and may have nucleic acids encapsulated in and lipid envelop outside the particle. For example, EVs are surrounded by a lipid membrane that contains cell membrane proteins very similar to an enveloped virus [5].

An ideal preparation process for such SLPPs must meet the need of impurity reduction to an acceptable level while maintaining the desired biological function of the product. However, the complex structures of the SLPPs which are often important for their biological activities are vulnerable to environmental stresses in downstream processing. Denaturation or losses of desired activities are frequently observed during various preparation processes [6, 7, 8], although these processes were developed to enhance the product quality and yield [9]. Such denaturation of SLPPs not only renders the process inefficient but also brings safety concerns. To avoid denaturation and achieve high yield more efficiently, rational design of the processing has been highly recommended for biopharmaceuticals in recent years [10]. The design should be based on a full understanding of the special characteristics of the product molecules. For SLPPs, several questions can be listed:

- How can we detect the SLPPs and their denaturation?
- Why are these particles denatured?
- What can we do about prevention of the denaturation?

**PRACTICAL APPLICATION**

Super large proteinaceous particles (SLPPs) such as virus, virus like particles, and extracellular vesicles have successful and promising applications in vaccination, gene therapy, and cancer treatment. However, the unstable nature and the complex particulate structure are challenges for their manufacturing and applications. Till now, we just begin to understand and have not yet obtained a complete map of which structural changes will affect biological activity of SLPPs, how to find these key micro structural changes, and how to effectively protect their biological activity during processing. This review highlights useful analytical techniques for characterization and stabilization of SLPPs in the process development and product formulations. These advanced analytical techniques will be helpful in obtaining deep insight into the mechanism related to denaturation of SLPPs, and more importantly, in seeking solutions to preserve their biological functions against deactivation or denaturation.

Experience has demonstrated that the conventional analytical tools for common proteins, such as SDS‐PAGE and ELISA, are neither enough nor efficient to study above topics. New analytical technologies will provide us an insight into the characteristics of SLPPs, and speed up the product development.

In this article, we will discuss challenges in manufacturing of SLPPs, and then highlight development and emerging applications of analytical techniques to address the critical issues in characterization and stabilization in process design for improved manufacturing and applications. This review is an update summary from the authors’ research and from the literatures [11, 12].

## 2 CHALLENGES IN MANUFACTURING OF SLPPS

From raw material to the final product, the manufacturing process of SLPPs generally includes expression, purification, and formulation. Whether the target particles have been successfully expressed and what the expression level is would determine the success of the follow‐up processes. Therefore, the quantification of bio‐active particles during cell culture is the first key of the whole production process. A rapid quantification of SLPPs can guide us to optimize cultivation conditions to improve the yield and subsequently cut the cost. Nevertheless, to quantify SLPPs is sometimes not easy. There are a large number of impurities in the medium including host cell proteins, host cell DNA, cell debris and residual proteins from medium. In the meantime, the yields of the target SLPPs are usually at low levels depending on the expression systems [13]. The characterization is difficult due mainly to the co‐production of particle‐related impurities including empty capsids, defective virus particles, free envelope proteins, aggregates and improperly assembled particles, and even EVs, which may possess similar compositions even size. These demand the analytical methods be able to sensitively and rapidly detect the target particles and differentiate them from impurities, especially the SLPPs related impurities.

The downstream purification is another bottleneck for manufacturing of SLPPs [14]. It requires not only the pure and concentrated proteins, but particles in correct structure to function their biological activity. The SLPPs related impurities usually have similar properties as SLPPs, which will probably be co‐purified. What's worse, various external pressures, including solution pH, temperature, high salts, shear force, and surface adsorption, will probably lead to denaturation of the naturally unstable SLPPs, further increasing the difficulty to achieve ideal recovery and purity. Although various SLPPs have been successfully purified with chromatography and membrane‐based separation techniques, there are many reports on denaturation or significant loss of SLPPs during these two methods [15]. For example, in the purification of hepatitis B surface antigen (HBs VLPs), a single ion exchange chromatography (IEC) or ultrafiltration step would lead to 20–40% loss in the total antigen recovery [16, 17], accompanied with the VLPs aggregation and dissociation. These failures expose the fact that we still do not fully understand the denaturation mechanisms and how to stabilize SLPPs during purification. Structural changes after purification can be easily studied by various techniques. However, in‐situ techniques that can monitor conformational changes taking place on the solid‐liquid interface during purification will be more informative. Sensor chip‐based technologies such as surface plasmon resonance (SPR) [18] and dual polarization interferometry (DPI) [19] have been widely applied for monitoring protein adsorption and molecular binding processed. By creating an adsorption surface mimicking the chromatographic media on chip surface, real time monitoring on the possible conformational changes of SLPPs on the solid‐liquid interface during adsorption would provide us deeper sight into the denaturation mechanism; thus, guide to more rational design of the process to accomplish a better yield and bio‐activity.

Due to the unstable nature and structural complexity, stable formulation of SLPPs is another critical factor ensuring their quality and potency during storage and transportation prior to application [6]. The stability of SLPPs can be evaluated through numerous methods that look at physicochemical or biological changes in a sample upon exposure to elevated pressures [6], and thermal stability is among the most important. Contrary to the extensive study of stability in solution, there are few studies paying attention to the stability for vaccine antigens after being adjuvanted. This could be hampered by the difficulties in characterization with interferences from adjuvants and low antigen concentration [20]. However, the structural alterations and subsequent loss of immune response by interaction with adjuvants have been found. It is gradually realized a rational formulation study should contain biophysical characterization of the particles, evaluation of stabilizers, and investigation of interactions with adjuvants [21].

**FIGURE 1:**
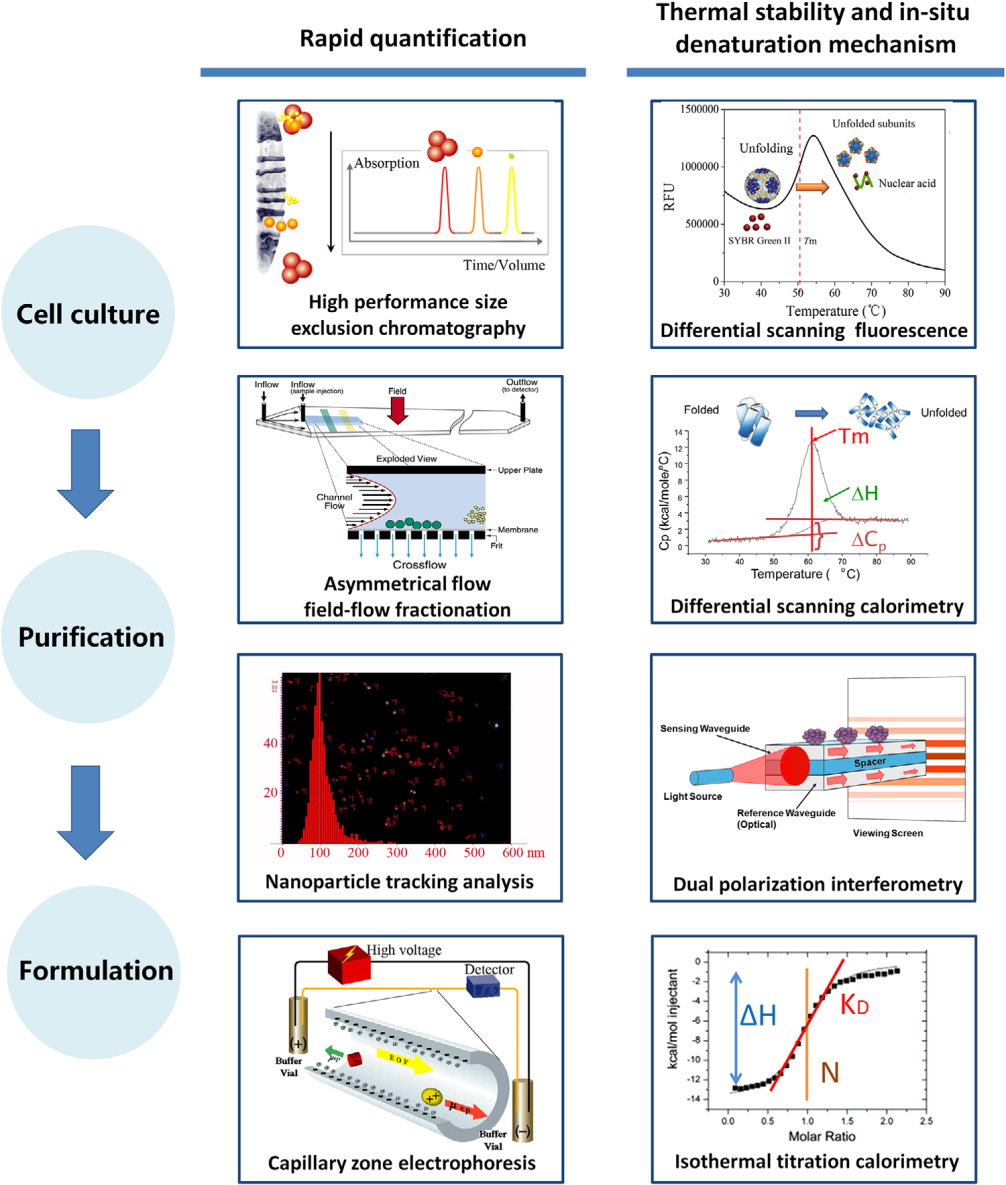
Some of typical analytical tools for rapid quantification, thermal stability, and in‐situ denaturation mechanism study of SLPPs in processing. Asymmetrical flow field‐flow fractionation (AF4) images are reproduced from [22]. Copyright 2011 Elsevier. Nanoparticle tracking analysis (NTA) image is reproduced from [23]. Copyright 2010 Society of Chemical Industry

To accomplish a higher level expression, more efficient purification with high purity and recovery, and more stable formulation for a better LLPs manufacturing, emerging applications of some typical analytical techniques will be described in the following sections (Figure 1).

## 3 ANALYTICAL TECHNIQUES FOR RAPID CHARACTERIZATION AND QUANTIFICATION OF SLPPS

### 3.1 High performance size exclusion chromatography

High performance size exclusion chromatography (HPSEC) is a non‐destructive technique separating particles based on a size dependent mechanism. It has been used for decades to analyze therapeutic proteins such as antibody. As for analysis of SLPPs, the main obstacle lies in the limited pore size and relatively low resolution of HPSEC matrix. SLPPs were often eluted within the void fraction, and it is difficult to discriminate between structural diversities of particles [24]. However, as the development of SEC technology, commercial analytical columns with pore size of matrix above 100 nm are available now, making it possible to analyze most large virus and VLPs as well as aggregates in the range of 10–100 nm [25]. The TSKgel G6000PWxl column, whose matrix diameter was determined from 20 nm to 2000 nm and the mean value of 250 nm, was reported to separate influenza A virus particle monomers, aggregates and fragments [26].

**FIGURE 2:**
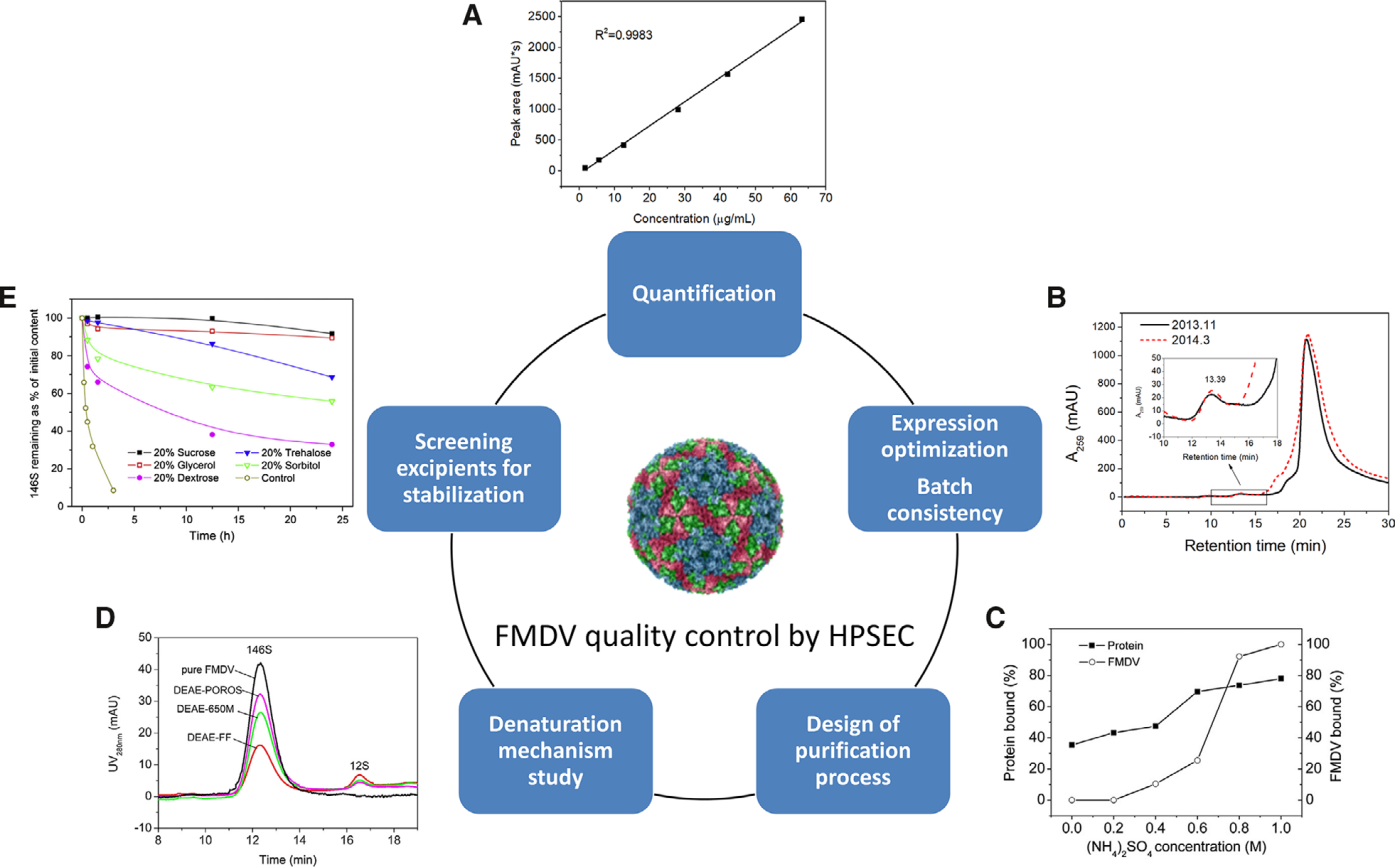
Applications of high performance size exclusion chromatography (HPSEC) in quality control and processing development of inactivated foot and mouth disease virus (FMDV) vaccine. (A) Calibration of peak area of FMDV in HPSEC analysis against concentration of FMDV standard for FMDV quantification. (B) Representative chromatograms from HPSEC analysis of FMDV vaccine preparation of different batches. (C) Optimization of hydrophobic interaction chromatographic purification of FMDV based on analyzing effect of (NH4)_2_SO_4_ concentration on FMDV bound efficiency as measured by HPSEC. (D) FMDV denaturation on different ion‐exchange media analyzed by HPSEC. (E) Thermal stability of FMDV at 45°C with addition of different excipients as measured by HPSEC. Figures (B–E) are reproduced with permissions from [29], [6], [7], and [32], respectively. Copyright 2015, 2015, 2017, 2017 Elsevier

Although HPSEC has been applied for SLPPs analysis, it is not paid much attention as a powerful tool for routine quality control of SLPPs as compared to other technologies. However, the popularity of HPLC equipment, simple and automated operation, and analytical columns of various specifications of HPSEC make it very attractive for product development or quality monitoring for research institutions, pharmaceutical companies and regulatory authorities. Successful examples are applications of HPSEC in the whole processing for inactivated foot and mouth disease virus (FMDV) vaccine, which is among the most important veterinary vaccines world wild. The intact FMDV, also called as 146S, has a poor stability that it is easily disassembled into smaller particles 12S with reduced immunogenicity [27]. Therefore, how to detect and stabilize the bio‐active 146S are the facing challenges for the vaccine production. The conventional ultracentrifugation is difficult to meet the requirement for rapid and robust detection [28]. ELISA is usually difficult to distinguish the intact virus from disassembled 12S or aggregates. Compared with these two methods, HPSEC could selectively detect 146S within 30 min [29]. In cultivation procedure, HPSEC successfully separated 146S from small and larger impurities in animal cell cultures, and quantified 146S yield according to its specific UV absorption (Figure 2A) [29]. The cultivation robustness of different batches was monitored by HPSEC chromatograms and FMDV yield (Figure 2B). During purification process design, the chromatographic media and operation conditions were rapidly screened as guided by 146S quantification as well as purity with HPSEC (Figure 2C). A hydrophobic interaction chromatography strategy was developed with a single step recovery of 92%, and total recovery and purity above 75 and 98%, respectively [7]. By detecting 146S and its dissociation products, the denaturation mechanism during IEC was investigated (Figure 2D) [8, 30]. For formulation study, the dissociating rate of 146S was quantitatively evaluated with HPSEC, which helped to efficiently screen for stabilizers both in solution and in adjuvants (Figure 2E) [31, 32]. Similar application of HPSEC can be applied to VLPs production [29]. By developing an interlaced procedure, the aggregates of several kinds of VLPs from 20 to 120 nm can be quantitatively measured within 3.1 min [25].

Coupling HPSEC with a multi‐angle laser light scattering (MALLS) detector further expands the application of HPSEC. MALLS detectors determine the weight‐average molar mass, average size by detecting the scattered light at multiple angles and extrapolating to 0° [11]. Therefore, HPSEC‐MALLS enables direct identification of SLPPs and aggregates during analysis by measuring the molar mass and size. Moreover, quantification according to number of particles can be obtained with MALLS without preparation of a calibration curve [25]. As the intensity of scattered light is proportional to the molar mass, it is highly sensitive to large particles like SLPPs. Quantification by MALLS reduced the lower limit of detection for HIV‐1 gag VLP by almost ten folds as compared to that of UV signal [24]. Few aggregates generated during processing that are difficult to detect by UV can be detected by MALLS [17]. What's more, by simultaneously coupling with differential refractive index (dRI) and UV detectors, HPSEC‐MALLS has been applied to characterize the relative capsid content (e.g. ratio of empty and full capsids) of AAV as claimed by the equipment manufacturer.

### 3.2 Asymmetrical flow field‐flow fractionation

Asymmetrical flow field‐flow fractionation (AF4) is the most established and commercially available field‐flow fractionation, which is a family of separation techniques suitable for the characterization of nano‐sized and micro‐sized systems [22]. Recently, AF4 has attracted increasing interest in the field of biomedicine owing to the utilization of an “open channel” void of stationary phase or packing material [33]. This avoids mechanical and shear stress that could cause entanglement and alterations in the native conformation of studied samples [33]. Different from HPSEC, whose separation range is limited by pore size of the stationary phase, AF4 can potentially separate molecules within a broad size range (∼10^3^–10^9^ Da; particle diameter from 2 nm to 0.5–1 µm) [34]; therefore, it is particularly suited for the analysis of SLPPs and aggregates.

The same as HPSEC, AF4 can be coupled to MALLS, dynamic laser light scattering (DLS), UV, and RI detectors. Wide applications of AF4‐MALLS have been reported for rapid assays on the size, size distribution, degradation, and aggregation of virus, VLPs and EVs. The AF4‐MALLS is applicable for analysis of both purified SLPPs and SLPPs in crude feedstock. When AF4 was applied for measuring the total particle counts of influenza virus and virus monomer/aggregate distribution in allantoic fluids of infected chicken eggs and in the supernatant of infected cultured cells [35], the results were comparable to other technologies including HPSEC‐MALLS, quantitative reverse transcription polymerase chain reaction (q‐PCR), median tissue culture dose (TCID50), and TEM, demonstrating AF4‐MALLS is a reliable technique for the detection of influenza viruses with excellent sensitivity and reproducibility. In comparison with these traditional virus quantitation methods such as TCID50 assays which take several days, or q‐PCR which requires labor‐intensive preparation of the RNA and standards, AF4‐MALLS allows detection for each sample within 1 h and eliminates tedious sample preparation steps. More importantly, it can provide critical information on the number, size distribution and aggregation state of vaccine candidate viruses at the same time. As the broad and feasible analysis range of AF4, it is also powerful in separation of fragments, oligomers, and aggregates in VLPs. The aggregation exposed to various solution conditions has been successfully studied by AF4‐MALLS for HBs VLPs [36] and murine polyoma virus (MPV) VLPs [37]. In vitro assembly is an important issue for VLPs products. With aid of AF4‐MALLS, the mechanism of self‐assembly of MPV VLPs was studied, and the recovery and homogeneity of the re‐assembled VLPs was efficiently improved [38].

Recently, AF4‐MALLS is getting attentions for characterizing the size distribution and particle number density of exosomes, which further exhibits the power of AF4 in separation of SLPPs. From the elution profile of the exosome sample displaying a broad size distribution from 23 to 113 nm (*R*_rms_) [39], exosome subpopulations (large exosome vesicles, 90–120 nm; small exosome vesicles, 60–80 nm) were identified by AF4‐MALLS, and the most abundant population of non‐membranous nanoparticles (∼35 nm) was discovered [40].

### 3.3 Nanoparticle tracking analysis

Nanoparticle tracking analysis (NTA) is a relatively new technology for simple and rapid characterization of size distribution and number density of particles with diameters generally from 30 to 1000 nm [41]. Similar to DLS, NTA is a non‐invasive and non‐separation method taking advantage of light scattering properties of particles. A video of the illuminated particles is captured and the movement of each particle is tracked frame by frame to obtain an average mean squared displacement. Then, the data are converted into the hydrodynamic diameter of the particle size [24]. The particle concentration is obtained when the tracked particles are counted and related to the sample volume [42]. As the NTA approach measures single particles simultaneously, the fastest detection time can be within 5 min [43].

Currently, NTA is most commonly used for characterization of exosome size and concentration derived from different cell types and enrichment methods [44]. It also has been applied for analysis of some virus and VLPs. The optimal set of NTA parameters enabled estimation of adenovirus, influenza virus, and lentivirus in virus harvest or purified samples over a large span of virus titters (10^7^–10^9^ part/mL) [23, 45, 46], and could be used as a sole or a complementary method for rapid virus recovery estimations in processing. NTA has been reported to directly count the HIV‐1 gag VLPs with about 10‐fold higher sensitivity than HPSEC‐MALLS [24]. As a particle‐based method, NTA is unable to distinguish between particles with similar size (infectious and non‐infectious particles for example). Two populations can only be resolved if their particle diameters differ by at least 1.5‐fold [39]. However, NTA can also track nanoparticles by their fluorescence if adequate wavelength excitation lasers and emission filters are applied [47], providing the possibility for selective analysis of SLPPs. Recently, the fluorescent NTA analysis was employed to detect and quantify human respiratory syncytial virus (RSV) by using fluorescent dye conjugated specific receptor which binds selectively to the virus envelope [47].

Despite the convenience to use, NTA has many drawbacks. For example, it is difficult for NTA to characterize poly‐dispersed samples. Compared to AF4‐MALLS, NTA tends to overestimate the particle concentration and have a relatively poor repeatability [43], especially for crude materials containing background particles [24]. Second, to make the NTA analysis possible, tedious dilution steps are usually needed for sample preparation to reach the rather narrow sample concentration gap between 10^7^ and 10^9^ particles per mL [42]. Another concern is that the selectivity of tracking and counting particles is highly influenced by several parameters that are adjustable by the user during analysis [45]. Consequently, it demands a skilled operator to obtain reliable results.

### 3.4 CE

The potential of CE in intact SLPPs analysis have been confirmed by successful examples of characterizing virus and VLPs by CZE, CIEF, and affinity CE in the last 2 decades [48]. CE analysis offers many advantages such as online monitoring, rapid detection, and low consumption of sample and reagents. CZE, the most popular CE for SLPPs analysis, has been regarded to be a potential option for the quality control of SLPPs in R&D of pharmaceuticals [49].

Like other colloidal particles, SLPPs carry charged or chargeable groups on their outer surface creating an electric double layer. Differential electromigration can thus be used to separate and characterize the intact SLPPs and related particles which exhibit distinct surface charges and zeta potentials depending on the buffer solution [50]. For example, CZE was reported to separate and determine the virus and subviral particles of human rhinovirus (HRV) [51] and HRV of different serotypes [52]. Binding with fluorescent dye and coupling with a LIF detector further enables higher detection sensitivity than UV detector and selective detection of different particles [53].

The ability for CZE to characterize SLPPs in crude preparation has been elevated. Okun et al. reported that the CZE allowed detecting serotypes HRV 16 and HRV 49 in crude and partially purified virus preparations [52]. However, Oita et al. found the complex salt and impurity composition in cell extracts very challenging to assess the poliovirus concentration [54]. In such condition, a sample preparation procedure using ultrafiltration or chromatography to purify and concentrate the SLPPs is necessary in order to allow injecting enough SLPPs to be above the detection limit and removing the interfering species [54].

The main challenge for CE analysis is the possible adsorptive interaction with the capillary wall leading to peak distortion or even loss of the analyte [51], which reduces its reproducibility and reliability. Detergents are often added to the background electrolyte (BGE) to reduce protein adsorption [55]. Another complication is the particular lability of SLPPs. The BGE has to be carefully chosen to minimize structural modifications of the SLPPs during electrophoresis [56]. In a recent work for intact adenovirus, CZE was validated to quantify the virus in samples from different manufacturing processes in the range of 0.5–1.5 × 10^11^ part/mL [57]. The total run time per sample was achieved within 5 min. The CZE method showed precision <5% RSD on concentration and accuracy of 90–110% after carefully optimizing the capillary type and the BGE composition to prevent virus adsorbing to the capillary. Moreover, the virus particle content obtained by CZE showed equivalency with other methods like qPCR. This example shows the power of CE in quality control of virus production at suitable operation condition.

## 4 TECHNIQUES FOR ANALYSIS OF THERMAL STABILITY AND DENATURATION MECHANISM OF SLPPS IN SOLUTION

### 4.1 Differential scanning calorimetry

Differential scanning calorimetry (DSC) is the only technique that directly measures the thermodynamics of intra‐ and inter‐molecular interactions stabilizing biological systems [58], which makes it popular for thermal stability study for various bio‐macromolecules. It determines the temperature and heat flow associated with material transitions as a function of time and temperature, and allows for the measurement of the temperature of protein unfolding (*T*_m_), the molar enthalpy change (Δ*H*), the molar entropy change (Δ*S*), and the change in the molar heat capacity (Δ*Cp*) accompanying a protein transition. These thermodynamic parameters are quite sensitive to the structural state of biomolecules. Any change in the conformation would affect the position, sharpness, and shape of transition(s) in DSC scans.

Subunit contact energies and free energy of subunit folding are two factors that contribute to particle stability of SLPPs [59]. Therefore, thermal denaturation of SLPPs can usually be separated into at least two distinct events, disruption of subunit contacts and protein unfolding. DSC can be a powerful tool to identify these key structural changes, especially the disruption process of the particle assembly.

**FIGURE 3:**
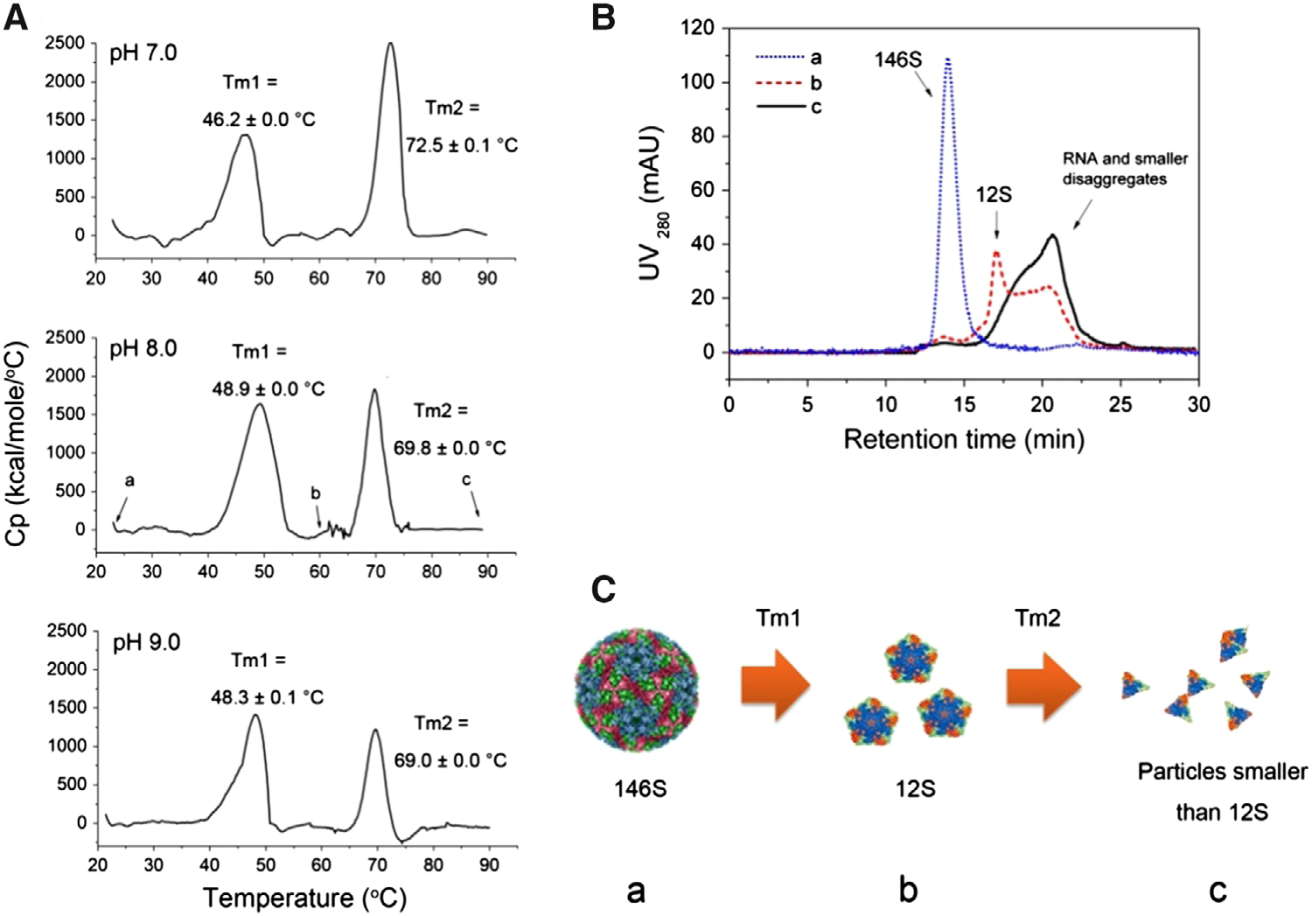
(A) The differential scanning calorimetry (DSC) thermograms of foot and mouth disease virus (FMDV) at pH 7.0, 8.0, and 9.0. (B) The high performance size exclusion chromatography chromatograms of FMDV at pH 8.0 and after being thermal scanned to (a) 23°C, (b) 60°C, and (c) 90°C, as indicated in the DSC thermogram of FMDV at pH 8.0. (C) The proposed mechanism for thermal dissociation of FMDV during these two transitions. Reprinted with permission from [32]. Copyright 2017 Elsevier

DSC has been used to evaluate the effect of additives and formulation composition on the thermal stability of FMDV [32]. With verification by HPSEC, the DSC detected two dissociation process of FMDV: the first transition is ascribed to the dissociation of intact virus into pentamer (*T*_m1_) which will result in significant loss of immunogenicity [60], and the second transition is the further dissociation of the pentamer 12S (*T*_m2_) (Figure 3). Therefore, by screening excipients increasing the *T*_m1_, the virus stability was significantly improved as verified by HPSEC and TEM [32]. It should be noted that significant structural alterations may still be present if the enthalpy of this structural change is not large enough to be detected at the measured concentration. Moreover, DSC results were sometimes not predictive of the excellent long‐term stability observed with other assays [61]. Therefore, DSC is often used in conjunction with other techniques (e.g. Raman, circular dichroism, light scattering) to gain more precise understanding of protein chemistry and structure [32].

Due to the sensitivity of DSC signals to different conformation, it can differentiate particles with similar properties such as SLPPs and related particles, different viral strains, and SLPPs that under through conformational changes during processing. Orlov et al. compared stability of virions, coat proteins (CP), aggregates of CP, and RNA‐free assembly of tobacco mosaic virus (TMP) by DSC [62]. When CP assembled into virion, its thermal stability was exceptionally increased for more than 30°C. The thermodynamic mode of virus unfolding is very specific for each viral strain of influenza virus, AAV, and poliovirus [59, 63], indicating DSC results can offer the possibility of precisely characterizing different viral strains. These results also provide guidance for selecting candidate stain for vaccine antigen and gene therapy. Product consistency of the hepatitis E virus (HEV) and human papilloma virus (HPV) VLPs vaccines has been evaluated by detecting the products from different lots. The *T*_m_ values obtained by DSC showed consistent values, indicating the antigen production process is robust and scalable during the manufacturing [61, 64, 65].

Understanding the thermal stability of SLPPs also can help us to design a purification procedure. Li et al. developed a purification method for hepatitis B core antigen (HBc) VLP with a pre‐heat treatment at 30 min heating at 60°C after they found the *T*_m_ of HBc VLP is as high as 96.25°C [66, 67]. About 75.30% of total protein was removed by forming precipitation while 89.8% of HBc VLP was recovered by this relatively low cost and simple operation.

### 4.2 Differential scanning fluorescence

Current DSC instrumentation requires relatively large amounts of protein sample. However, the molecular weights of SLPPs are usually beyond several million Dalton and the concentration is generally below 0.1 mg/mL; therefore, the extremely low molarity in the samples will probably fail to induce a detectable DSC signal [31]. Meanwhile, high throughput techniques are more appealing for SLPPs product development. Differential scanning fluorescence (DSF), which is performed in 96‐well plates using a real‐time polymerase chain reaction (RT‐PCR) instrument, begins to attract attention in SLPPs analysis for its high sensitivity and high throughput. DSF measures the fluorescence of an extrinsic probe that is sensitive to the denaturation on the protein surface during heating. Until the protein has completely unfolded, the fluorescence will reach a maximum. Thus, the midpoint of the change in fluorescence is taken to correspond to the transition temperature (*T*_m_). Afterwards, there is often a decrease in the fluorescence signal that is attributable to thermal quenching and/or aggregation of the protein [68].

DSF can be used across a broad solution conditions for stability study; thus, has been proven robust and powerful in excipients screening for the formulation design [69]. Comparison of DSF and DSC showed there is a correlation between the transition temperatures determined by the two techniques [59]. Sometimes, DSF is able to reflect small changes in SLPPs structures during processing that cannot be detected by other techniques. Due to this advantage, DSF has been evaluated to be a reproducible, fast, and low‐cost method to ensure batch to batch consistency in manufacturing facilities and academic laboratories for AAV [70]. The fluorescent fingerprints not only enabled serotype identification [71], but also provided information on sample homogeneity, particle concentration, and buffer composition [70].

The SYPRO Orange is the most popular dye used in DSF which binds to hydrophobic sites externalized during unfolding. The DSF thermogram of inactivated FMDV with SYPRO Orange as dye exhibited two *T*_m_ around 50 and 70°C, respectively [69], in accordance with DSC analysis [32]. The detection also can be conducted by using dyes that are sensitive to the changes in the membrane order or intercalating dyes that can detect capsid disruption by binding to the viral nucleic acid [69]. With SYBR green II as a reporter for RNA, the dissociation of FMDV have been successfully detected [31] with a much higher sensitivity than with SYPRO Orange. Using SYBR Green II as fluorescent dye enables detection of FMDV as low as 5 µg/mL, while 30 µg/mL of FMDV still cannot give an evident signal by SYPRO Orange [31]. Another advantage of SYBR Green II is its tolerance to viral samples with low protein purity, and therefore it is suitable for quality control in different processing procedures [31]. On the contrary, large amount of protein impurities will disturb both DSC and DSF using SYPRO Orange [70]. However, using nucleic acid dye only can detect one *T*_m_ referring to capsid dissociation of virus, and further denaturation of subunits cannot be detected [31]. Therefore, some researchers recommend the use of two dyes simultaneously would provide a picture of the changes in the particles [72].

## 5 ANALYTICAL TECHNIQUES FOR IN‐SITU STUDY OF DENATURATION ON THE SOLID‐LIQUID INTERFACE DURING SLPPS PURIFICATION

### 5.1 Dual polarization interferometry

DPI is an emerging optical biosensor utilizing a waveguide interferometer and two polarization beams (TE and TM) that split and propagate through the waveguide [19]. Any changes caused by the adsorption of molecules to the waveguide surface will interfere with the light propagation. By addressing the signals of the two beams simultaneously, the thickness, layer density and mass of an equivalent uniform adsorbed layer at the sensor–liquid interface can be monitored in real time [73]. That enables DPI as an ideal tool for study of protein adsorption at different surfaces and for evaluation of changes in protein structure at the solid–liquid interface [74, 75].

Recently, DPI was employed to study the adsorption and conformational change of HBs VLPs on ion exchange surface at three different pHs, aiming to have an insight into the disassembling and loss of activity during IEC [76]. By creating an ion exchange surface on chip surface, the conformational changes of HBs VLPs during adsorption to the surface were monitored in real time for the first time. According to the data determined by DPI, the HBs VLPs formed a 33.1 nm bi‐layer at pH 5.0, and formed monolayers packed with significant spreading at pH 7.0 and pH 9.0, whose thickness was only 13.8 nm and 6.6 nm, respectively. Combined with HPSEC analyses of VLPs structural integrity and changes in antigen activity after IEC process, the possible disassembling mechanism of HBs VLPs during chromatography was discussed. The protein‐surface interactions resulting in intense multi‐point binding were considered the primary mechanism that induce the spreading and subsequently disassembling of HBs‐VLPs in desorption process. Unfavorable “lateral” protein–protein interactions may also contribute to the most serious disassembling of HBs‐VLP and the lowest antigen recovery found at pH 5.0.

The study by DPI directly showed the specific behaviors of SLPPs at solid‐liquid interfaces as large and soft particles. More studies can be conducted by modifying the sensor chip with other ligands to stimulate the chromatographic or membrane adsorption surfaces.

### 5.2 Isothermal titration calorimetry

Isothermal titration calorimetry (ITC) is an accurate method for estimating the total heat change in various biological reactions, providing a complete thermodynamic profile of the molecular interaction without labeling. The measurement allows direct determination of binding constants (*K*a), reaction stoichiometry (*n*), Δ*G*, Δ*H*, and Δ*S* [77]. In ITC study, reactive materials can be studied not only in solution, but also in the form of particulate suspensions, since the turbidity or the color of the samples has no influence on the measurement. Furthermore, the determination has no molecular weight restriction. These characteristics make ITC possible to study protein adsorption at solid‐liquid interfaces such as chromatographic media and particulate adjuvants.

Knowledge of the binding mechanism of proteins at the solid‐liquid interface provides valuable insights into the separation and unfolding of proteins, which offer valuable guidelines for designing experimental conditions in chromatographic separation. To date, detailed thermodynamic characteristics have been reported on hydrophobic interaction chromatography (HIC), immobilized metal ion affinity chromatography (IMAC), and IEC [78]. In the experiment, the adsorbent is suspended in the equilibrium buffer solution and placed in the sample cell. Then it is titrated by protein solution prepared in the equilibrium buffer solution. The output signals are collected as power vs. time and are integrated and quantified by the amount of bound protein, which is obtained from the equilibrium binding isotherms, to give the enthalpy change of adsorption.

**FIGURE 4:**
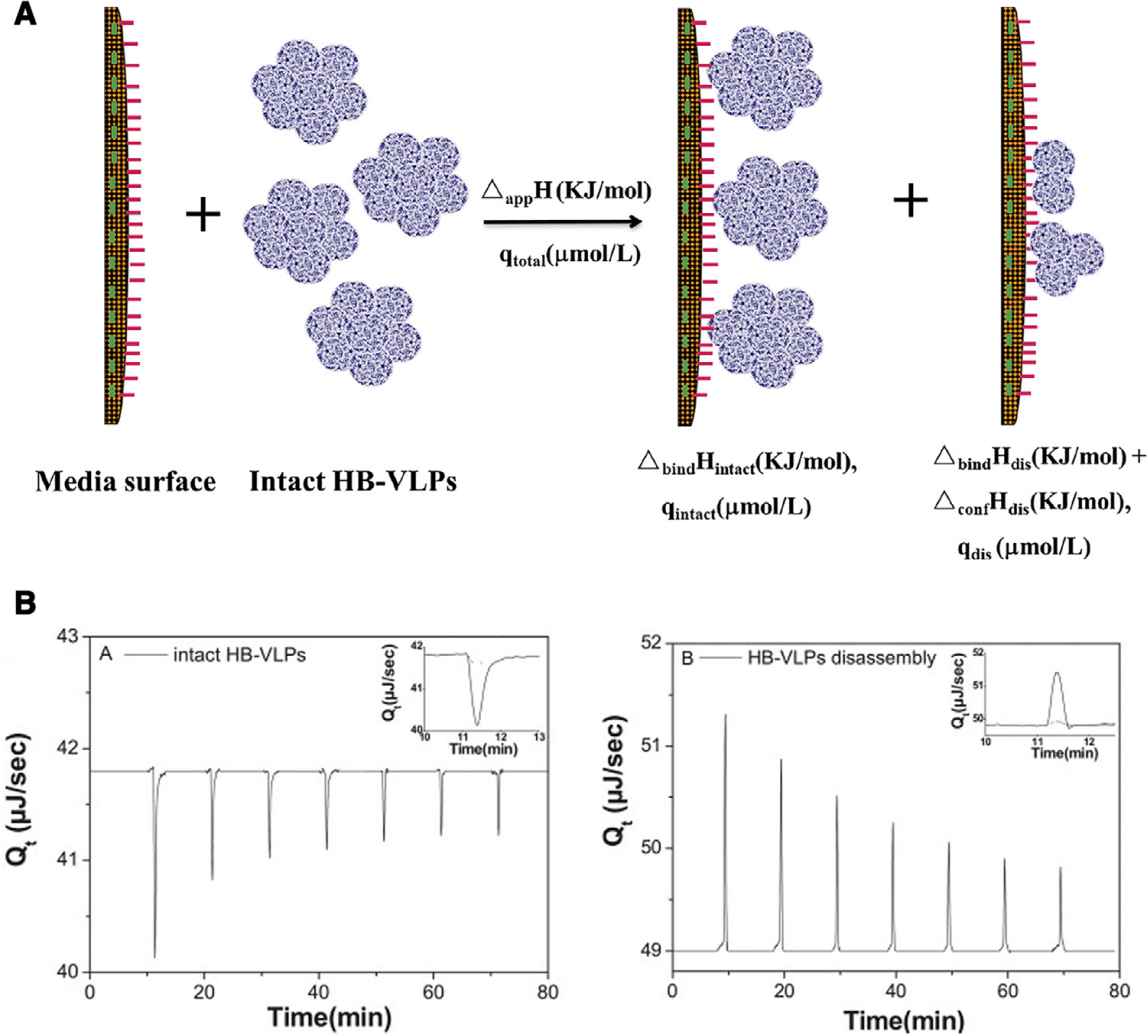
Isothermal titration calorimetry (ITC) study of the adsorption and disassembling of intact hepatitis B surface antigen (HBs) virus like particles (VLP) on gigaporous IEC media surface. (A) Schematic illustration of adsorption. Certain amount of HBs VLPs are adsorbed in intact formation, *q*_intact_ (mol/L), and the molar enthalpy change associated to this binding is referred as Δ_bind_ *H*_intact_; meanwhile, certain amount of HBs VLPs are disassembled and adsorbed simultaneously on the solid–liquid interface in the formation of disassembled HBs VLPs, *q*_dis_ (mol/L), and the molar enthalpy change associated to its binding and the enthalpy change accompanying the disassembling of HBs VLP are referred as Δ_bind_ *H*_dis_ and Δ_conf_ *H*_dis_, respectively. (B) Representative ITC thermograms of titration of intact HBs VLP and HBs VLP disassembled formation into 0.02 g‐media/mL DEAE‐POROS suspension (a kind of ion exchange media with ligand density 0.222 mmol/mL) at 25°C. Reprinted with permission from [79]. Copyright 2015 Elsevier

By measuring adsorption enthalpies of partially unfolded protein and resolving the enthalpy change accompanying the conformational change in presence of denaturants, the thermodynamic mechanism of protein unfolding on the interface can be studied by ITC. Nevertheless, information relating to thermodynamics of SLPPs adsorption is still lacking by now. Yu et al. extensively investigated the thermodynamic mechanism of the adsorption and disassembling of VLPs on liquid‐solid interface during its adsorption on gigaporous IEC media surface for the first time (Figure 4) [79]. The IEC media with high ligand density led to significantly increased irreversible disassembling of HBs VLPs and consequently low antigen activity recovery. Therefore, the apparent adsorption enthalpy of HBs VLPs (Δ_app_ *H*) was considered containing three terms: (1) the intrinsic molar enthalpy change associated to the binding of intact HB‐VLPs (Δ_bind_ *H*_intact_), (2) the intrinsic molar enthalpy change associated to the binding of HB‐VLPs disassembled formation (Δ_bind_ *H*_dis_), and (3) the enthalpy change accompanying the disassembling of HB‐VLPs (Δ_conf_ *H*_dis_) (Figure 4A). The Δ_conf_ *H*_dis_ can be calculated according to the other parameters which can be obtained by ITC (Figure 4B). The negative Δ_conf_ *H*_dis_ suggested an enthalpy‐driven process for the disassembling of HBs VLPs during IEC. Moreover, as ligand density increase, it became more negative, which was in agreement with the findings from chromatography experiments that higher ligand density led to more serious disassembling of HBs VLPs.

Another application for biothermodynamic analysis by ITC is antigen‐adjuvant interactions in a vaccine formulation. Similar to the studies in bio‐separation, the ITC approach can be helpful to identify interaction mechanisms and explore the optimum conditions for protein‐adsorbent interactions. However, few studies were reported for SLPPs antigens to date.

### 5.3 In‐situ thermal stability study on solid‐liquid interface by DSC and DSF

As introduced previously, DSC and DSF are powerful thermal stability study techniques. In fact, they not only allow detection for subjects in solution, but also in the form of particulate suspensions. The turbidity of the samples is not expected to interfere with the measurement since the DSC detects heat changes [8], and the detection is usually conducted at 90° or from the top of the multi‐well plates as for DSF. This property implies these two methods can be applied for in‐situ stability study for proteins on solid‐liquid interface.

DSC and DSF have been successfully applied for investigating the effects of adjuvants on structures of proteins including SLPPs [63]. It was reported adsorption onto aluminum salt adjuvants may result in structural alterations and less thermally stable for both monomeric protein like lysozyme and SLPPs such as virus and VLPs [80]. However, it does not always lead to conformational changes. The possible effect of adsorption to aluminum adjuvant has been monitored using DSC for HPV VLPs, and no significant shift in *T*_m_ was observed, indicating that the adsorption had no effect on the structural integrity of the products [61, 65]. In another study for FMDV, DSF was employed as a versatile method for in‐situ and high throughput study of the thermal stability of FMDV in different adjuvants [30]. The DSF enabled screening for pH conditions and excipients in both aluminum adjuvant and oil emulsions, and the results were verified by DSC and HPSEC. The *T*_m_ of FMDV was found diverse under different solution conditions and adjuvants, suggesting the stabilization or destabilization by interaction with adjuvant is correlated to the kind of adjuvant and formulation conditions. That highlights the importance of formulation study with presence of adjuvant. Since formulation is the last step of processing, only by defining the final antigen state can we really understand what nature can lead to better immunity in vivo.

**FIGURE 5:**
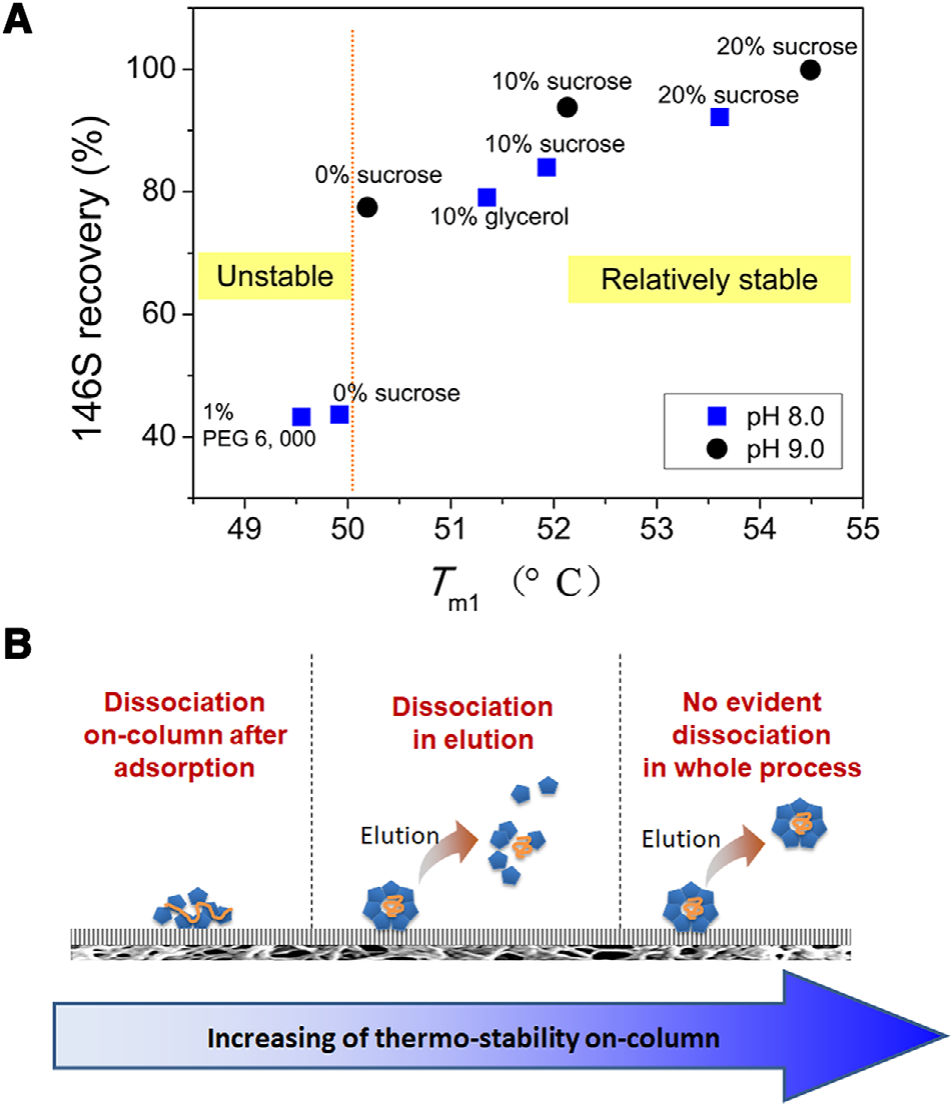
Relationship between thermal stability of foot and mouth disease virus (FMDV) and dissociation during ion exchange chromatography (IEC). (A) Correlation of FMDV recovery in IEC in presence of different excipients and at pH 8.0 and 9.0 and the corresponding *T*_m1_ on IEC media surface determined by DSC. (B) Illustration of proposed relationship between FMDV thermal stability on‐column and dissociation during IEC. Reprinted with permission from [30]. Copyright 2020 Elsevier

The thermal stability of SLPPs adsorbed on chromatographic media was recently reported on studies of FMDV dissociation during IEC [8, 30]. Interaction between inactivated FMDV and the DEAE media would result in decrease of thermal stability of the virus as revealed by the decreased *T*_m_ in DSC, which explained the dissociation and low recovery. Moreover, the *T*_m_ exhibited correlation with the dissociation on column that both the virus recovery and *T*_m_ decreased as the pore size of media decreased [8]. When FMDV were absorbed on DEAE‐POROS, whose pore size is about 7.6‐fold of the size of FMDV, less contact was formed between the FMDV and binding sites, so that dissociation was avoided and a higher stability and a recovery of 94.62% was obtained. A study by DSC at different pH and with several excipients further verified the positive dependence of on‐column stability with recovery [30] (Figure 5A). Three different dissociation processes of FMDV in IEC depending on the stability con‐column were illustrated: dissociation upon adsorption, dissociation during elution, and no dissociation in the whole process (Figure 5B). Such in‐situ DSC analysis provides a perspective to understand the denaturation of SLPPs assemblies during chromatography, and also supplies a strategy to improve assembly recovery.

## 6 OTHER ANALYTICAL TECHNIQUES

In addition to the techniques discussed above, MS, NMR, electron and atomic force microscopy have contributed to our understanding of the structure of SLPPs. Besides various physicochemical techniques, immunochemical methods including ELISA, biosensor analysis, RIA, and non‐labelled immunoassays are also important for characterization of SLPPs. These methods are valuable since the integrity of structures is often important for in vivo bio‐activities, although a direct link between in vivo biological activities and structures has not been fully understood [20]. Sometimes the denaturation leads to subtle conformational changes that are difficult to detect by physicochemical techniques. In such condition, immunochemical methods may give us more sophisticated information.

## 7 CONCLUDING REMARKS

The increasing development of emerging therapies and the demand for new biotherapeutic medicines has challenged the biopharmaceutical industry. Proteinaceous particles with super large size and complex composition showed their own unique characterization and production challenges. Till now, we just begin to understand and have not yet obtained a complete map of which structural changes will affect biological activity of SLPPs, how to find these key micro‐structural changes, and how to effectively protect their biological activity during processing. A rapid and rational SLPPs development is supposed to combine different analytical techniques based on different principles for rapid quantification, thermal stability, and in‐situ denaturation mechanism study as outlined in Figure 1. Analytical techniques for rapid characterization and quantification of SLPPs based on their particulate properties of samples are listed and compared in Table S1, which include HPSEC‐MALLS, AF4‐MALLS, NTA and CZE. Proper techniques can be selected according to their characteristics and the sample's properties. In‐situ and thermal dynamic analytical methods, such as DPI, ITC and DSC, can provide more information on assembly/disassembly mechanism of SLPPs. Successful application of an optimal combination of these different techniques have been fully demonstrated, for example, in production process of inactivated FMDV, which include utilizing HPSEC for rapid characterization and quantification for the bio‐active antigens (Figure 2), DSC for thermal stability and dissociation mechanism study of antigens both in solution and during purification [7, 30, 32], and DSF for high throughput formulation study in adjuvants [31]. Correlating physicochemical results to in vitro or in vivo biological activity analyses is considered to be the future trend to guarantee a high quality, safety, and efficacy of SLPPs, but the studies are still lacking.

## CONFLICT OF INTEREST

The authors have declared no conflict of interest.

## Supporting information

Supporting information
