# Supplementary material for: Characterization and stabilization in process development and product formulation for super large proteinaceous particles
Source: Eng Life Sci. 2020 Jul 19;20(11):451–65. doi: 10.1002/elsc.202000033 (PMC7645648; doi:10.1002/elsc.202000033)
Supplement: Supplementary file 1 — Supporting information [file ELSC-20-451-s001.docx]

**Supplementary Information**

Review

**Characterization and stabilization in process development and product formulation for super large proteinaceous particles**

Yanli Yang^1^

Zhiguo Su^1^

Guanghui Ma^1^

Songping Zhang^1^

^1^State Key Laboratory of Biochemical Engineering, Institute of Process Engineering, Chinese Academy of Sciences, Beijing 100190, PR China

**Correspondence:** Prof. Songping Zhang (spzhang@ipe.ac.cn). State Key Laboratory of Biochemical Engineering, Institute of Process Engineering, Chinese Academy of Sciences, Beijing 100190, PR China.

**Supporting Table**

**Table S1** Comparison of techniques for rapid characterization and quantification of SLPPs based on particulate properties.

| Techniques | HPSEC-MALLS | AF4-MALLS | NTA | CZE |
| --- | --- | --- | --- | --- |
| Size range (nm) | 10-250 | 2-1000 | 30-1000 | Depending on electromigration |
| Time cost | ~30 min | Depending on separation | 5 min to 1 h | Depending on separation |
| Concentration range (particles/mL) | > 2.1 ×10^9^ | > 10^6^ | 10^7^-10^9^ | > 5×10^10^ |
| Applied range | Cell culture, purification, formulation | Cell culture, purification, formulation | Purified samples | Cell culture, purification, formulation |
| Size information | Yes | Yes | Yes | No |
| Additional fluorescence measurement | No | No | Yes, Enables higher sensitivity and specificity | Yes, Enables higher sensitivity and specificity |
| Ref. | [1-3] | [4, 5] | [6-8] | [9] |

HPSEC, high performance size exclusion chromatography; MALLS, multi-angle laser light scattering; AF4, asymmetrical flow field-flow fractionation; NTA, nanoparticle tracking analysis; CZE, capillary zone electrophoresis.

**Supporting references**

[1] Steppert, P., Burgstallera, D., Klausberger, M., Tover, A., et al., Quantification and characterization of virus-like particles by size-exclusion chromatography and nanoparticle tracking analysis, *J. Chromatogr. A* 2017, *1487*, 89-99.

[2] Vajda, J., Weber, D., Brekel, D., Hundt, B., et al., Size distribution analysis of influenza virus particles using size exclusion chromatography, *J. Chromatogr. A* 2016, *1465*, 117-125.

[3] Yang, Y. L., Li, H., Li, Z. J., Zhang, Y., et al., Size-exclusion HPLC provides a simple, rapid, and versatile alternative method for quality control of vaccines by characterizing the assembly of antigens, *Vaccine* 2015, *33*, 1143-1150.

[4] Heider, S., Metzner, C., Quantitative real-time single particle analysis of virions, *Virology* 2014, *462*, 199-206.

[5] Bousse, T., Shore, D. A., Goldsmith, C. S., Hossain, M. J., et al., Quantitation of influenza virus using field flow fractionation and multi-angle light scattering for quantifying influenza A particles, *J. Virol. Methods* 2013, *193*, 589-596.

[6] Gross, J., Sayle, S., Karow, A. R., Bakowsky, U., et al., Nanoparticle tracking analysis of particle size and concentration detection in suspensions of polymer and protein samples: Influence of experimental and data evaluation parameters, *Eur. J. Pharm. Biopharm*. 2016, *104*, 30-41.

[7] Du, S. F., Kendall, K., Morris, S., Sweet, C., Measuring number-concentrations of nanoparticles and viruses in liquids on-line, *J. Chem. Technol. Biot*. 2010, *85*. 1223-1228.

[8] Papanikolaou, E., Kontostathi, G., Drakopoulou, E., Georgomanoli, M., et al., Characterization and comparative performance of lentiviral vector preparations concentrated by either one-step ultrafiltration or ultracentrifugation, *Virus Res.* 2013, *175*, 1-11.

[9] van Tricht, E., Geurink, L., Backus, H., Germano, M., et al., One single, fast and robust capillary electrophoresis method for the direct quantification of intact adenovirus particles in upstream and downstream processing samples, *Talanta*, 2017, *166*, 8-14.
